# Cystic brain metastasis is associated with poor prognosis in patients with advanced breast cancer

**DOI:** 10.18632/oncotarget.12176

**Published:** 2016-09-21

**Authors:** Bing Sun, Zhou Huang, Shikai Wu, Lijuan Ding, Ge Shen, Lei Cha, Junliang Wang, Santai Song

**Affiliations:** ^1^ Department of Radiotherapy, Affiliated Hospital of Academy of Military Medical Sciences, Beijing 100071, China; ^2^ Department of Science and Technology, Academy of Military Medical Sciences, Beijing 100071, China; ^3^ Department of Breast Cancer, Affiliated Hospital of Academy of Military Medical Sciences, Beijing 100071, China

**Keywords:** breast cancer, brain metastases, cyst, whole-brain radiotherapy, stereotactic radiosurgery

## Abstract

**Purpose:**

Brain metastasis (BM) with a cystic component from breast cancer is rare and largely uncharacterized. The purpose of this study was to identify the characteristics of cystic BM in a large cohort of breast cancer patients.

**Results:**

A total of 35 eligible patients with cystic BM and 255 patients with solid BM were analyzed. Three factors were significantly associated with an increased probability of developing cystic lesions: age at diagnosis ≤ 40 years, age at BM ≤ 45 years, and poor histological grade (*p* < 0.05). Patients with cystic metastasis were also characterized by a larger metastasis volume, a shorter progression-free survival (PFS) following their first treatment for BM, and poor overall survival after BM (*p* < 0.05). Multivariate analysis further demonstrated that local control of cystic BM was only potentially achieved for HER2-negative primary tumors (*p =* 0.084).

**Methods:**

Breast cancer patients with parenchymal BM were reviewed from consecutive cases treated at our institution. Cystic BM was defined when the volume of a cystic lesion was greater than 50% of the aggregated volume of all lesions present. Clinicopathologic and radiographic variables were correlated with development of cystic lesions and with prognosis of cystic BM.

**Conclusions:**

This study shows that cystic BM from breast cancer, a special morphological type of BM, had worse prognosis than the more commonly observed solid BM. Younger age and low tumor grade were associated with the development of cystic lesions. Further comprehensive research and management of cystic BM are warranted to improve its poor prognosis.

## INTRODUCTION

Brain metastasis (BM) is a common intracranial tumor and one of the principle causes of death in cancer patients. It is currently known that BM predominantly originates from lung cancer, breast cancer, and other malignancies, and the prognosis of patients with BM from breast cancer has been extensively characterized [1–3]. Moreover, the survival time of patients with breast cancer BM has been found to be the longest. The incidence of BM has been escalating due to an increase in patient cancer survival and advances in brain screening technologies. It is estimated that approximately 10–30% of patients with breast cancer will develop BM [4]. Therefore, many studies have focused on the treatment and outcome for patients with breast cancer BM [5–7].

For BM from breast cancer, the typical morphological characteristics include a solid lesion with irregular margins and peri-tumoral edema. Clinically, the presence of a cystic component has been associated with poor response and outcome, yet a lack of clear clinical data exists. There are several studies that have focused on local treatment and response of cystic cerebral metastasis in small cohorts with different primary malignancies [8–14]. However, the characteristics and prognosis of cystic brain metastases has not been explicitly elucidated in large population studies.

In this study, we analyzed 290 patients with BM that originated from breast cancer and present the properties of the cystic metastases detected. Thus, the aims of this study were to: (a) identify the characteristics of cystic BM development and its risk factors, and (b) analyze the treatment and prognosis of patients with cystic BM.

## RESULTS

### Patient characteristics

A total of 290 eligible patients were analyzed and their characteristics and outcomes are listed in Table 1. Briefly, most patients of the present cohort were premenopausal women with a median age of 43 years at diagnosis of HR-positive (57.2%), luminal subtype (54.5%), and stage II (65.2%) tumors. The median DFS was 23.8 months, and the median time from a DFS event to diagnosis of BM was 44.6 months. The median follow-up period after diagnosis of BM was 28.0 months (95% CI, 23.7–32.3).

**Table 1:** Characteristics and factors affecting the development of cystic brain metastases

Characteristic	All patients n (n/290)	Patients with solid lesions n (n/255)	Patients with cystic lesions n (n/35)	*p*-value
Median age at diagnosis, y (min–max)	43 (23–76)	44 (23–76)	38 (25–57)	0.013^Δ^
Median age at BM, y (min–max)	48 (26–79)	48 (26–79)	44 (28–61)	0.025^Δ^
Nodal status				0.423
Negative	110 (37.9)	98 (38.4)	11 (31.4)	
Positive	180 (62.1)	157 (61.6)	24 (68.6)	
Histological grade#	136 (46.9)	119 (46.7)	17 (48.6)	0.032
I	4 (2.9)	4 (3.4)	0 (0.0)	
II	112 (82.4)	101 (84.9)	11 (64.7)	
III	20 (14.7)	14 (11.8)	6 (35.3)	
Clinical stage (I-II *vs.* III-IV)				0.356
I	31 (10.7)	30 (11.8)	1 (2.9)	
II	189 (65.2)	165 (64.7)	24 (68.6)	
III	42 (14.5)	35 (13.7)	7 (20.0)	
IV	28 (9.7)	25 (9.8)	3 (8.6)	
HR status†				0.247
Positive	166 (57.2)	149 (58.4)	17 (48.6)	
Negative	122 (42.1)	104 (40.8)	18 (51.4)	
HER2 status				0.574
+ (IHC+++ / FISH+)	106 (36.6)	94 (36.9)	12 (34.3)	
- (IHC-/+ / FISH-)	161 (55.5)	139 (54.5)	22 (62.9)	
Unknown or IHC++	23 (7.9)	22 (8.6)	1 (2.9)	
Subtype of primary tumor*				0.413
Luminal	158 (54.5)	142 (55.7)	16 (45.7)	
Triple-negative	67 (23.1)	56 (22.0)	11 (31.4)	
HER2 positive	55 (19.0)	48 (18.8)	7 (20.0)	
Median DFS, m (95% CI)	23.8 (0.0–232.4)	23.8 (0.0–232.4)	24.5 (0.0–122.0)	0.871
Median TTBM, m (95% CI)	44.6 (0.0–353.3)	44.7 (0.0–353.3)	40.2 (0.0–158.8)	0.645
Median follow-up time after BM, m (95% CI)	28.0 (23.7–32.3)	28.1 (24.1–32.1)	25.7 (6.9–44.5)	0.452

# Tumor grade was determined by two pathologists from our institution by using the Nottingham combined with histologic grade (Elston-Ellis modification of Scarff-Bloom-Richardson grading system) criteria. Tumor grades for 154 (53.1%) patients were unknown/uncertain.

† HR status of the primary tumor was unknown for two patients.

* Classification of biological subtypes was based on 2011 St. Gallen international expert consensus. Ten patients (3.4%) with uncertain expression of HER-2 and/or Ki67 were not classified into any specific subtype. All receptors results were detected from primary tumor.

Δ *p*-values were calculated using categorical variables of age at diagnosis (≤ 40 y vs. > 40 y) and age at BM (≤ 45 y vs. > 45 y), respectively.

Abbreivations: DFS, disease-free survival; FISH, fluorescence *in situ* hybridization; HR, hormone-receptor, the status of estrogen receptor (ER) and/or progesterone receptor (PR); HER2, human epidermal growth factor receptor 2; IHC, immunohistochemistry; BM, brain metastases; TTBM, time to brain metastasis, calculated from the date of a DFS event to the date of BM diagnosis; m, month; y, year; 95% CI, 95% confidential interval.

### Risk factors associated with the development of cystic metastasis

Of the present cohort, 35 patients (12.1%) had cystic BM. The risk factors related to the probability of developing cystic metastasis were analyzed (Table 1) and an age at diagnosis ≤ 40 years, an age at BM ≤ 45 years, and poor histological grade were significantly associated with the development of cystic lesions (*p* < 0.05 in each case). Therefore, patients with cystic tumors were younger in age and their tumors were more aggressive compared with the patients with solid BM. However, tumor subtype, the most prognostic factor, was not found to have any association with the presence of cystic lesions.

### Characteristics and outcome of patients with cystic metastases

**Figure 1:**
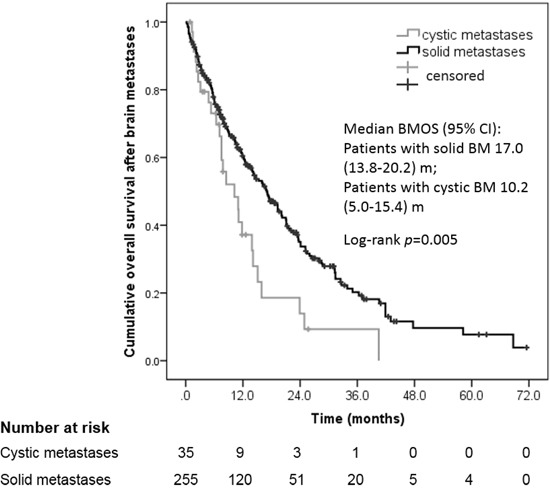
Cumulative overall survival (OS) after diagnosis of cystic versus solid brain metastases

For the present cohort, the mean 1-year BMOS was estimated to be approximately 44%, with the median BMOS and OS periods being 15.3 months and 75.4 months, respectively (Table 2). Most of the patients (52.4%) had oligometastatic disease (1~3 metastases), while most of the patients (60.0%) with cystic lesions had non-oligometastic disease (≥ 4 lesions), yet there were no significant differences found between these two groups (*p* = 0.117). The patients with cystic lesions had a heavy tumor burden with a mean aggregated volume of 25.8 cm^3^ versus 9.2 cm^3^ for the patients with solid lesions (*p* < 0.001). Furthermore, the median PFS period for the cystic lesion group was 4.2 months (95% CI 1.0–8.0) versus 8.2 months (95% CI 6.7–9.7) for the solid lesion group after the first treatment for BM (*p* < 0.001). Consequently, the prognosis for the patients with cystic metastasis was poorer than that for the solid metastasis group (10.2 months *vs.* 17.0 months for BMOS, *p* = 0.005, Figure 1).

**Table 2:** Characteristics and outcome of brain metastasis cases

Characteristics	All patients n (n/290)	Patients with solid lesions n (n/255)	Patients with cystic lesions n (n/35)	*p*-value
No. brain parenchyma lesions (%)				0.117
Oligometastases (1–3 lesions)	152 (52.4)	138 (54.1)	14 (40.0)	
Non-oligometastases (≥ 4 lesions)	138 (47.6)	117 (45.9)	21 (60.0)	
Volume of all BM/cystic lesions*, cm^3^				< 0.001#
Min~max	0.0–132.2	0.0–87.1	0.6–132.2	
Median	5.2	4.4	14.0/12.9	
Mean	11.2	9.2	25.8/24.1	
Median PFS of first treatment for BM, m (95% CI)	8.0 (6.7–9.3)	8.2 (6.7–9.7)	4.2 (1.0–8.0)	< 0.001
Death (%)	191 (65.9)	165 (64.7)	26 (74.3)	0.262
Median BMOS, m (95% CI)	15.3 (12.6–18.0)	17.0 (13.8–20.2)	10.2 (5.0–15.4)	0.005
Median OS, m (95% CI)	75.4 (68.5–82.2)	75.7 (69.1–82.3)	63.1 (52.6–73.7)	0.196

* For aggregated volume of cystic lesions;

# analyzed using independent samples t-test.

Abbreviations: BM, brain metastasis; BMOS: overall survival after BM; OS: overall survival; PFS: progression-free survival; m, month; 95% CI, 95% confidential interval.

### Treatment for cystic metastases

Thirty-three patients were included in the analysis of tumor control time (PFS), while two patients were excluded because they did not receive treatment for severe symptomatic BM. Seventeen (51.5%) of the 33 patients were treated only with whole brain radiotherapy (WBRT), 10 (30.3%) patients received local modalities (including stereotactic radiosurgery, surgery, aspiration, and/or Ommaya reservoir), and 6 (18.2%) patients received WBRT combined with local modality as a first treatment.

Univariate Cox regression analysis for PFS of first treatment for cystic metastasis was performed with the clinical and treatment variables listed in Table 3. HER2-positive was associated with a worse outcome of cystic metastasis (*p* < 0.1), while there was no relationship between the aggregated volume of lesions and PFS (*p* = 0.745). The other clinical variables and treatments examined were not associated with PFS (*p* > 0.1). In the multivariate analysis, only HER2-negative primary tumors were potentially predicted to achieve better local control of cystic BM (*p* = 0.084).

**Table 3:** Cox regression analyses of clinical variables and PFS for cystic brain metastasis

Covariate	Comparison	*p*-value	Hazard ratio (95% CI)
Univariate analysis			
HER2 status of primary tumor	- vs. +	0.041	0.40 (0.17–0.96)
HER2 status of metastatic tumor#	- vs. +	0.121	0.45 (0.16–1.24)
Subtype of primary tumor	Non-triple-negative *vs.* Triple-negative	0.166	0.54 (0.22–1.30)
Subtype of metastatic tumor#	Non-triple-negative *vs.* Triple-negative	0.132	0.44 (0.15–1.28)
Aggregate volume of BM, cm^3^	> 14.0 *vs.* ≤ 14.0	0.745	1.13 (0.54–2.36)
Treatment		0.287	
	Local modality *vs.* WBRT + local modality	0.132	2.53 (0.76–8.47)
	WBRT *vs.* WBRT + local modality	0.447	1.55 (0.50–4.81)
	WBRT *vs*. local modality	0.271	0.61 (0.26–1.47)
Multivariate analysis			
HER2 status of primary tumor	- vs. +	0.084	0.44 (0.17–1.12)

CI, confidence interval

# Receptor status of metastatic tumor was obtained for 23 patients.

## DISCUSSION

Recently, several studies have focused on the treatment of cystic metastatic lesions that arise from different malignancies (Table 4) [8–14]. Histologically, most of these lesions derived from lung cancer and these had the highest incidence of BM and the most cystic BM patients published [8, 9, 15]. Patients with non-small-cell lung cancer (NSCLC) harboring anaplastic lymphoma kinase (ALK)-rearrangements that were treated with crizotinib have also presented with cystic BM [16–19]. Traditionally, cystic BM has been associated with a poorer response and prognosis. However, the clinical characteristics and prognosis of this specific type of lesion arising from specific malignancy have not been comprehensively reported. Therefore, to our knowledge, the current study is the first to investigate the cystic features of BM in a large cohort of breast cancer patients, and to identify risk factors for cystic BM formation and prognosis.

**Table 4:** Summary of studies of patients with cystic brain metastases underwent radiotherapy

Publication	Total pts	Cyst BM definition	No. of cystic lesions (total lesions)	No. of pts with BC	Treatment for cystic BM	Mean cyst volume pre-treatment, ml (range)	Mean radiation dose, Gy (range)	Local control	Prognostic factors
Yamanaka et al., 2006 [14]	22	Large cystic BM	28 (103)	11	Ommaya reservoir placement followed by GKRS	40.1 (27-58)	14.9 (8-20)	67.9% at mean f/u of 11.5 m	-
Franzin et al., 2008 [12]	30	A maximum of 4 lesions and at least 1 cystic lesion	33 (81)	6	Stereotactic drainage followed by GKRS	21.8 (3.8-68)	19.5 (12-25)	91.3% with median f/u of 9 m	Extension of the extracranial illness, RPA classification, male sex and different tumor types predicted survival
Park et al., 2009 [11]	24	Large cystic BM	25 (−)	7	Stereotactic cyst aspiration, with or without Ommaya reservoir insertion, then GKRS	23.2 (7.9-100.9)	20.2 (13-25)	54.2% with median f/u of 13.1 m	-
Higuchi et al., 2012 [10]	25	Large cystic BM	25 (−)	7	Stereotactic aspiration followed by GKRS at the same day	20.3 (8.0-64.2)	21.6 (10-23)	76.2% at median f/u of 11 m	-
Ebinu et al., 2013 [8]	73	A cystic component	111 (111)	11	GKRS	3.3 (0.1-23)	21 (15-24)	63.0% at 1y	Lung primary predicted local control
Jung et al., 2014 [9]	24	Patients with cystic BM received aspiration and GKRS	29 (−)	1	Cyst aspiration and GKRS	18.6 (8-72.3)	16 (14-20)*	58.6%	No significant factor related to OS
Lee et al., 2015 [15]	28	Patients harboring at least one cystic BM underwent GKRS	37 (−)	5	GKRS (aspiration of cysts >10 ml in 8 patients)	25.1 (10.9-57.6)#	16.6 (13-22)	82.3% at 1y	Controlled primary tumor for survival; prescription dose (>15 Gy) for local control

All of the publications listed are retrospective studies. - Indicates the specific number was not provided or applicable.

# The volume of 8 cystic metastases before aspiration.

* Median prescription dose.

Abbreviations: GKRS, gamma knife radiosurgery; RPA, recursive partitioning analysis; pts, patients; BC, breast cancer; Gy, Gray; m, month; y, year; f/u, follow-up.

Cystic BM may include cystic/necrotic tumors with thin/thick enhancing walls with less/medium perifocal edema [20]. Several studies have focused on large cystic BM. These studies mainly included cysts with a volume > 10 ml, with the mean volume ranging from 20–33 ml (Table 4) [10–12, 14, 15]. Compared with other published inclusion criteria for cystic BM [8, 12], the definition of cystic BM used in the present study is probably the strictest in order to represent the predominant property of BM in individual patients and to represent the proximal status of cystic BM. In addition, the definition allowed a range of cyst volumes to be included, which differs from most of the literature that has studied large cystic BM. Consequently, the proportion of cystic type BM in the present cohort was relatively high (12.1%), although its actual incidence may be < 10% since almost all of the eliminated cases had solid brain metastases or only meninges lesions.

Our retrospective study found that an age at diagnosis of breast cancer ≤ 40 years, an age at BM ≤ 45 years, and low histological grade correlated with an elevated risk of cystic lesion development in advanced patients. However, other important predictors, especially for phenotype and subtype, were not associated with cyst development. Our results also indicated that the presence of a cystic component was more likely in patients with more aggressive disease. Accordingly, we hypothesized that a cystic composition including noncellular cystic fluid or a necrotic component may be produced due to rapid metastasis growth. In a study by Yeh et al., cystic necrotic BM was significantly associated with triple-negative breast cancer (TNBC) (with cystic-necrotic BM diagnosed in 9/12 of the patients with TNBC) [11]. In the early studies that were conducted, cyst formation appeared to occur due to disruption of the blood-brain barrier and the subsequent edematous process [21, 22]. However, the mechanism(s) and inherent biological differences between the tumor cells associated with solid versus cystic lesions have not been elucidated [23]. Furthermore, the gene signatures of cystic tumor cells located in the cyst wall and in mural nodules have not been investigated. Therefore, a better understanding of these aspects is needed to prevent the development of metastases, while also facilitating the early detection and treatment of metastases that have developed.

Here, the cystic metastasis group had a larger metastasis volume than the solid metastasis group. Furthermore, the PFS period following the first treatment for BM was shorter in the former group than in the latter group. Accordingly, a significant difference was observed in BMOS as a function of cystic lesion type. Therefore, we attempted to identify important treatment and prognostic factors for the prognosis and tumor control of cystic lesions. The patients with HER2-positive or triple-negative tumors were prone to a shorter PFS period following the first local modalities. Yet, other factors, including therapeutic methods and lesion volume, did not appear to affect median PFS. It is known that many factors affect treatment decisions, and these factors can include control of extracranial disease, performance status, the need for surgery or cyst aspiration, volume and location of a lesion, multifocality, BM-related symptoms, and extent of edema. Table 4 lists the prognostic factors of survival or local tumor control that were indicated in four studies. However, it is difficult to make conclusions from these data due to the small sample size for each study, as well as the differences in enrollment criteria, histology, and treatment among the studies. Furthermore, different treatment strategies can result in different responses and varying extents of tumor control. Therefore, our present findings regarding the features and outcome of cystic BM should be confirmed in future studies, and local control that is achieved with different treatment procedures for cystic metastasis should be further analyzed in larger samples.

There were limitations associated with the present study. First, the sample size for the cystic BM group was relatively small, and this is consistent with the low incidence of cystic BM. Second, the information regarding histologic grade and phenotype of the primary and metastatic tumors was incomplete due to the retrospective nature of this study. Third, the complex treatment for BM in the present cohort was not analyzed in detail. An unfavorable factor that affected treatment response was the requirement for a high radiation dose to achieve local benefit due to the insensitivity of the cystic component. Regarding the confounding factors and inconsistent treatments, radiation dose, re-irradiation data (available for 14/35 patients that received additional irradiation due to *in situ* and/or distant recurrence of metastases in the brain), and other factors that might be associated with treatment response were not analyzed. Thus, the present findings remain to be confirmed in a larger population.

In summary, the present study focused on cystic BM, a special morphologic type of BM, in order to investigate the risk factors, characteristics, and prognosis of this BM in a large population of breast cancer patients. The results obtained indicate that a younger age and low tumor grade are two factors that were associated with the development of cystic lesions. Moreover, cystic BM was associated with a significantly larger volume, a shorter PFS period, and a worse prognosis than solid BM. In addition, only HER2-negative primary tumors were potentially related to a longer PFS period. Therefore, in order to improve the poor tumor control and prognosis that currently characterize cystic BM from breast cancer, a specific subset of the affected patient population should be further studied. It is anticipated that these results will be of great interest to clinicians that are responsible for the comprehensive management of cystic metastases in advanced patients with the goal of improving quality of life and prolonging patient survival.

## PATIENTS AND METHODS

### Patients

We reviewed a retrospective database of 385 consecutive patients diagnosed with breast cancer and BM that were treated at our hospital between January 2008 and October 2014. A total of 319 advanced patients were selected based on the availability of their integrated clinical and radiographic information, treatment, and follow-up data. In 29 patients, meningeal metastases without a parenchyma lesion were observed, and these patients were excluded as previously reported [3]. Therefore, a total of 290 eligible patients were enrolled and analyzed for this study. The brain parenchyma metastases were categorized as solid lesions or cystic lesions, and were confirmed by gadolinium contrast-enhanced magnetic resonance imaging (MRI) with or without clinical symptoms and pathology. Radiographic data for the response assessment of BM were obtained from baseline and subsequent follow-up appointments. Treatment response was evaluated by using Response Evaluation Criteria in Solid Tumor (RECIST) criteria (version 1.1). The clinical variables examined included: age at diagnosis, age at BM, tumor staging, and tumor subtype among others. This retrospective study was approved by the Human Investigations Committee of the Affiliated Hospital of Academy of Military Medical Sciences. All patients provided written informed consent to permit their medical records to be used for research purposes.

**Figure 2:**
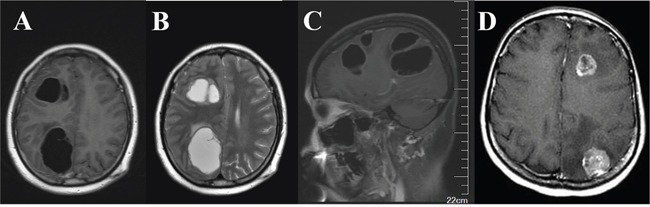
Typical characteristics of cystic versus solid brain metastases observed in MRI images Axial T1-weighted (A) and T2-weighted (B) post-contrast MRI of right frontal and parietal cystic metastases with appearance of hypointensity **A.** and hyperintensity **B.** and surrounding edema. **C.** Sagittal T1-weighted post-contrast imaging. **D.** Axial T1-weighted MRI of solid lesions.

Cystic BM, a special type of BM, was defined as the presence of a cystic/fluid component which can be determined by the radiologic appearance of hypo/hyper intensity on T1/T2-weighted MRI imaging [8, 20]. In general, the volume of the cystic lesions was also greater than 50% of the aggregated volume of all of the lesions. Solid BM was another type of brain parenchyma lesion that was identified in the cohort examined. Figure 2 presents the typical characteristics of cystic versus solid BM that were observed in MRI images.

### Treatment for cystic metastases and radiographic data

The important factors that guide treatment selection include tumor volume, tumor number, lesion site, patients' symptoms, Karnofsky performance status (KPS), and extracranial disease. Treatment for cystic metastases was largely determined by the participation of physicians, neurosurgeons, radiation oncologists, and radiation physicists with comprehensive consideration of the above variables. First-line treatment for BM was included in the response analysis. Axial images from pre-treatment and post-contrast MRIs with gadolinium contrast-enhanced T1-weighted sequences were imported to the Eclipse treatment planning system (version 7.3, Varian Medical Systems, Palo Alto, CA, USA) for delineation of metastases.

### Statistical analysis

Events for the calculation of disease-free survival (DFS) included local-regional relapse and distant recurrences. Progression-free survival (PFS) for first-line local treatment was defined as the time between the start of treatment for BM and confirmed intracranial progression, last follow-up date without progression, or breast cancer-related death. Overall survival after BM (BMOS) was estimated from the time of BM diagnosis to breast cancer-related death or last follow-up. The median follow-up after diagnosis of BM was calculated using reverse Kaplan-Meier method [24]. At the last follow-up on July 1, 2015, there were 191 deaths and 26 patients were lost to last follow-up. Among the latter patients, the follow-up time after diagnosis of BM was longer than 24 months for 10 of the patients, whereas it ranged from 1 to 22.2 months for the remaining 16 patients (median time, 9.5 months).

All statistical analyses were performed by using SPSS 13.0 software (SPSS, Chicago, IL, USA). A binary logistic regression model was used to determine the relationship between the clinical variables and cystic metastases. Cox regression analyses were used to identify independent variables associated with PFS in all patients. Variables with a *p*-value < 0.1 in the univariate analysis were entered into a multivariate model. According to the Kaplan-Meier method, the survival curves were compared with a log-rank test. A two-tailed *p-*value less than 0.05 was considered statistically significant.
